# Cerebral Palsy Link to Sensorimotor System, Cognition, Emotion and Nociplastic Pain

**DOI:** 10.3390/children12060702

**Published:** 2025-05-29

**Authors:** Wolfgang Laube, Mathilde Sengoelge

**Affiliations:** Department for Health Sciences, Medicine and Research, Faculty of Health and Medicine, Center for Health Sciences and Medicine, A-3500 Krems, Austria

**Keywords:** sensorimotor developmental delay, chronic pain, cerebral palsy, deconditioning, cognition, emotions, movement

## Abstract

This narrative review provides an overview of the link between the sensorimotor system, cognition, emotion and pain syndromes in persons due to deconditioning or delayed sensorimotor development, then applied to persons with cerebral palsy (CP). The brain damage that occurs before, during or even after birth pathophysiologically alters the structure and subsequent function of the sensorimotor function, which is inseparably linked to cognition, emotion, behavior and pain. This damage results in a functional developmental disorder that also affects the structure and function of the neuromatrix in a graded manner due to chronic deconditioning. It is the basis for both primary and secondary chronic degenerative diseases. This leads to an increasing prevalence of chronic pain syndromes, which may be very high in persons with CP. Thus, CP is both a disposing factor and a causal factor for nociplastic pain, defined as persistent pain arising from altered nociception without evidence of tissue or somatosensory damage. Therapy interventions are crucial to optimize movement, cognition and emotion, as well as pain reduction in persons with CP.

## 1. Introduction

The aim of this narrative review is to provide an overview of the link between the sensorimotor system, cognition, emotion and pain syndromes in persons due to deconditioning or delayed sensorimotor development, then specifically applied to persons with cerebral palsy (CP) that involves both factors. The sensorimotor system consists of the peripheral and central nervous systems and musculature. Depending on the level of movement abilities, it is responsible for the following: all movements with a specific cognitive performance; non-movement-specific cognitive and emotional brain functions that shape learning ability, competencies, and overall behavior; and the function of pain inhibition and pain modulation. Impairments in the sensorimotor system may occur in an individual due to several factors: due to deconditioning, due to sensorimotor developmental delay, and, most severely, due to cerebral damage before, during or even after birth. These impairments influence and determine movement and may lead to chronic degenerative diseases with chronic pain as a symptom. If symptoms worsen, nociplastic pain syndromes may develop. Nociplastic pain is defined by the International Association for the Study of Pain as persistent pain that arises from altered nociception, despite no clear evidence of actual or threatened tissue damage causing the activation of peripheral nociceptors, or evidence for disease or lesion of the somatosensory system causing the pain [1]. This demonstrates the intensive networking of the neuromatrix and the fact that both functional developmental delay due to deconditioning and structural developmental delay due to CP are each a predisposition to, and at the same time, a factor in the occurrence of cognitive and emotional behaviour modifications and pain syndromes.

In this review, the focus is on persons with CP. Pre- and perinatal or even postnatal damage to the central nervous system before the age of two is responsible for CP. The etiology is multifactorial and includes genetic, infectious or pollutant-related and more frequently vascular and ischemic factors. Damage-related inflammatory processes are also involved in its development [2,3]. Premature babies are disproportionately at risk due to the vulnerability of the vascular system [4,5] and have generally lower levels of functional and structural brain-related proteins [6]. The symptoms and consequences are highly variable depending on the site of damage in the brain and the extent of the damage. Early childhood brain damage disrupts the brain’s ability to control movement and maintain posture and balance. This is termed CP, whereby the term “cerebral” refers to the brain and “palsy” refers to the loss or impairment of motor function. CP implies a disruption of the sensorimotor and corticomotor networks and their interactions, leading to a loss of selective motor control, gross motor function, postural control and notably spasticity. The spasticity is due to the muscle volume being massively reduced and the quality of the contractile properties being reduced, thus shortening the muscles. In addition, the ability to regenerate is reduced and inflammatory processes take place.

The development of the sensory systems with their central processing structures and the correct timing of early childhood reflexes are essential elements in the maturation of sensorimotor function. Sensorimotor development is the “biological stimulator” for the development of the highest brain functions, as there is no sensorimotor development without cognitive and emotional functions and performance. For example, from a sensorimotor perspective, the vestibulo-ocular and vestibulo-spinal reflexes ensure that the positioning of the head is controlled and thus provide the prerequisite for the visual system to be able to depict the environment in a stable manner and for the brain to be able to process the information [7,8]. It also enables the body to straighten up, allowing for essential functional elements of sensorimotor coordination and postural regulation to develop balance and precision of movement. Infants who cannot maintain balance and are placed in passive and premature upright positions in the first year of life were shown to have impaired quality of postural, coordination, and reflex functions in adolescence [9]. In addition to the importance of movement, there is the interconnection between the sensorimotor system and the neuronal networks for memory and emotion [10]. The brain does not have a pain center, but there are pain components involving the highest functional levels: sensory-discriminative, cognitive-evaluative and affective-emotional. The development of sensorimotor functions is coupled with pain inhibition and pain modulation to form a functional unit in healthy individuals [11]. Pronounced early childhood damage to the brain, combined with early childhood sensorimotor developmental delays, can be regarded as factors for the development of nociplastic pain syndromes. The very high prevalence of chronic pain in persons with CP speaks for this [12,13].

The following sections of this review describe the interrelationships between brain structure, sensory-motor function, cognition, emotion and pain syndromes.

## 2. Link Between Impaired Sensorimotor Function Due to Deconditioning and Pain

**Figure 1:**
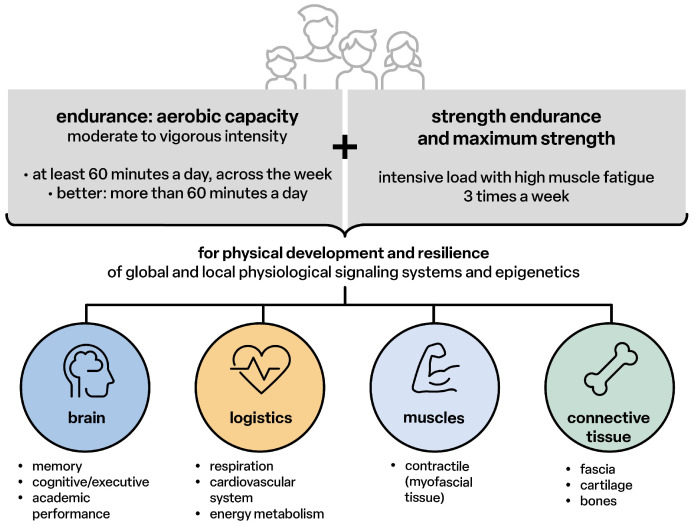
Physical activity for children, adolescents 5–17 years [14].

The healthy development of children and adolescents requires at least one hour of moderate to intensive physical activity per day [14] in order to:

- Allow the genetic potential of physical development to become “fully” effective;
- Enable all peripheral body structures and the nervous system with the brain as the highest level of behavior, communication and the integral cooperation of all structural and functional levels into a healthy, resilient, powerful and resistant functional state;
- Maintain the structures and functions throughout the entire lifespan or delay the ageing process by continuing physical activities for at least 1.5 to 3.0 h per week (Figure 1).

**Figure 2:**
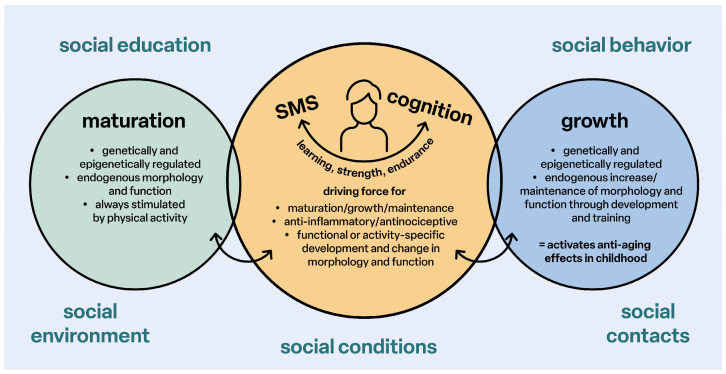
Stimulation of maturation and growth; SMS: sensorimotor system.

Structurally effective physical activities for movement skills (sensorimotor coordination), endurance and strength are the essential driving forces for sensorimotor and cognitive-emotional and social development, maturation, the anti-inflammatory status of all tissues and organs, healthy growth and the reduction of the risk of developing primary chronic degenerative diseases in every age group (Figure 2).

**Figure 3:**
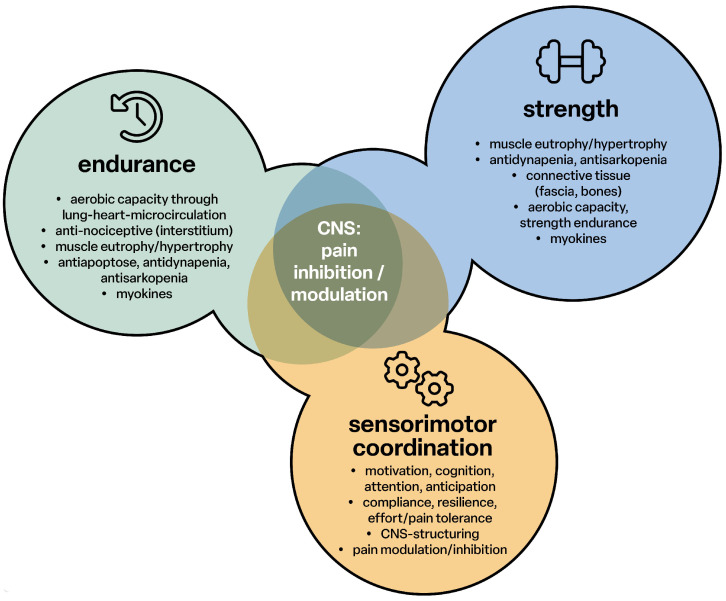
Pain inhibition through endurance, strength and sensorimotor coordination; CNS: central nervous system.

Endurance, strength training and sensorimotor coordination support pain inhibition and modulation in the central nervous system. The cerebral functions are supported via the myokines to develop and promote memory function and train resistance to exertion and pain (Figure 3).

Thus, a well-developed and healthy brain structure is responsible for sensorimotor, cognitive-emotional performance, anti-nociceptive function and reduces the risk of nociceptive development. The basis for this is the neuromatrix, an intensive mutual networking of all unconscious and conscious structural levels of the brain.

**Figure 4:**
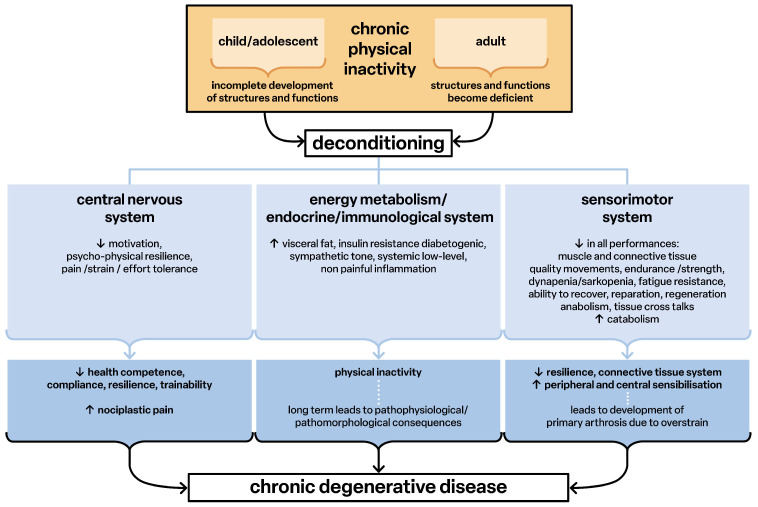
Deconditioning as factor for development of chronic degenerative disease. Chronic physical activity leads to deconditioning and this over time leads to chronic degenerative disease.

Lack of exercise leads to deconditioning, defined as the structural and functional weakness of all tissues and organs. This weakness has pathophysiological characteristics and causes generalized low-grade inflammation (Figure 4). This non-painful inflammatory process is the basis of all chronic degenerative diseases (diseasome of physical inactivity) [15], including the inflammatory process in the brain [16] that leads to adverse pathological changes in functioning and performance (Figure 4).

In deconditioning, all functions of the sensorimotor system are impaired. The communication of the tissues via their signaling substances and genetic information (exosomes) [17,18,19] promotes the mutual pathophysiological structuring and thus the further development of diseases (Figure 4). The overall result is the development of nociplastic pain syndromes [20,21,22] in which the reported pain can no longer be explained by peripheral findings of tissue damage or inflammation [23]. Thus, pathophysiological developmental processes may occur in primarily structurally and functionally healthy people because of chronic physical inactivity.

## 3. Link Between Delayed Sensorimotor Development and Cognition, Emotion

Sensorimotor development begins in the 18th to 20th week of pregnancy and after birth, and early childhood reflexes are the basic sensorimotor stimuli for structural and functional maturation and growth. This is the time of development of the sensory systems, whereby kinaesthetic and visual perception are linked and form the basis for the development of the cerebral body schema. The body schema or body representation is not a perception or a map of the body in the brain, but a construct of the interpretation and integration of multisensory information [24,25]. It is an idea of one’s own body. The brain needs this idea for the correct sensory identification of the body compartments in relation to each other and in space (own-body recognition), for the recognition of relations to the environment and as an essential basis for the regulation of movement [26,27]. Individuals with delayed sensorimotor development are people with retained primitive reflexes. Children, adolescents and adults with retained primitive reflexes may have a coexisting neurobehavioral disorder or “learning disability”. This has been identified in persons with ADHD, autism, Tourette’s, dyslexia, or other neurobehavioral disorders [28].

Primitive neonatal reflexes are the basic developmental stages of postural regulation. They ensure head control, which in turn is the prerequisite for oculomotor function for stable visual perception, body erection and sensorimotor coordination, and the reflexes link motor performance with cognitive development. Maturational and developmental delays impair both sensorimotor skills and cognition. Children become particularly noticeable at school age in terms of learning, behavior, language development and academic performance. Developmental delays in sensorimotor skills in a very mild form can cause similar disorders of cognition and emotional behavior as early childhood brain damage. 

## 4. CP and Link to Damage to All Brain Functions

Infantile CP is a non-progressive, heterogeneous condition that develops during pregnancy, birth or in the neonatal period, depending on the time and extent of brain damage (ischemia, neurotoxicity). The initial damage to the brain often occurs in the early fetal developmental phase [29]. Up to 90% of premature infants have a lesion of the periventricular white matter (ischemia). In children born at term, lesions are preferentially cortical and/or subcortical in the thalamus and the basal ganglia, which represent the second most common disorder pattern [30]. The findings of cerebral structural and functional connectivity (networking) vary between the clinical subtypes of CP: unispastic (approximately 30%), bilateral spastic (approximately 55%) and dyskinetic CP (approximately 7%), which together account for over 90% of cases, with ataxic CP approximately 4% present [31]. The wide variety of clinical pictures in CP could be caused by damage-related inflammatory reactions. In the analysis of cerebrospinal fluid (*n* = 28, age 9.7 ± 4.4 years, minimum 4 years, maximum 23 years), markers of neuroinflammatory mediators, endocrine hormones and nociceptive neuropeptides were found as potential characteristics of the clinical outcome. There are significant relationships between the individual parameters with very high correlation coefficients (*p* ≤ 0.001). The association patterns vary depending on the birth dates: timely, early or extremely early. The correlations of the parameters TNF-α and substance P separate premature and extremely premature births. Overall, these results reveal relationships between the functional systems of arousal (orexin A [regulation of sleep–wake behavior]), inflammation (TNF-α), inhibition of inflammation (IL-1ra) and neuronal arousal (substance P). It can therefore be assumed that the damage to the white matter triggers an immune-mediated inflammatory cascade and characterizes the clinical picture [3]. As a result of the damage to the developing brain, children and adults with CP are characterized by differently pronounced sensorimotor, cognitive and emotional deviations in behavior, language, learning ability and intelligence compared to healthy individuals.

## 5. CP and Link to Sensorimotor Function and Cognition

**Figure 5:**
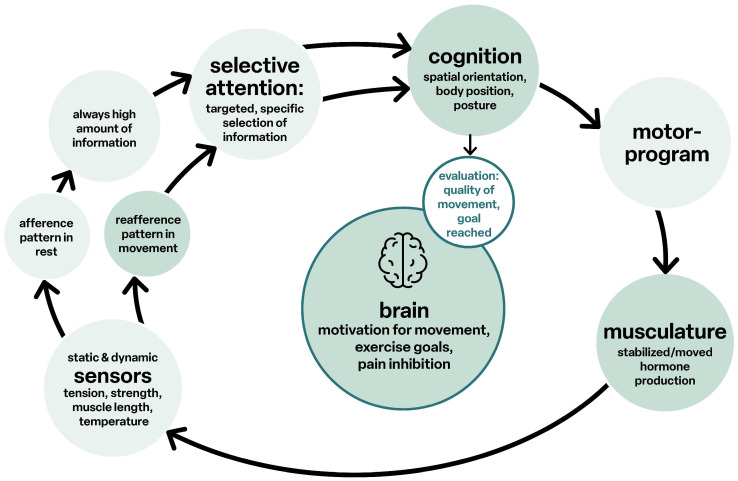
Cognition and movement.

Sensorimotor function is the distinctive feature of life, as its function is linked to movement and cognition, all together in a functional unit [32], see Figure 5.

The structural networks of the brain form a unit, and complex tasks are realized through the integrative interconnection of the various relevant sub-areas to form “functional networks”. Cerebral disorders of sensorimotor function, as in CP, are therefore always also deviations in cognition and vice versa.

The structures of the sensorimotor system [33] are affected on both the afferent and efferent sides. Mailleux et al. [34] emphasize that the integrity of the sensory and motor subsystems of both cerebral hemispheres should always be considered diagnostically in order to explain the variability of clinical sensorimotor findings. Even with clinically unilateral CP, it is very likely that bilateral brain damage is present in a very large proportion of children [35,36]. There are very heterogeneous changes in the gray matter and very great similarities between primary and secondary damage to the basal ganglia. Primary lesions of the periventricular white matter cause secondary changes in the gray matter of the thalamus, the basal ganglia and the cortex. The sensory pathways and connections, the capsula interna, the cortico-spinal pathway systems and the cortico-bulbar tract show losses in integrity (number of nerve fibers, fiber arrangement, degree of myelination, axonal damage, volume) compared to children who are physiologically developed. In the case of severe damage, the connectivity of the thalamus with the parietal and occipital lobes is similarly impaired. As a result, the visual-spatial function and motor performance of the upper limbs are even more severely affected. The structure of the thalamus–somatosensory cortex information pathway is also damaged [37].

In children with unilateral CP, the volumes of the deep gray matter do not differ between those resulting from a cortical and deep gray matter lesion or a periventricular white matter lesion, and the thalamus is smaller on both sides. Compared to healthy individuals, the volume reduction is unilateral, and the cortical and subcortical lesion is additionally combined with widespread cortical changes in all lobules [36]. Individuals with diplegic spastic CP have, with few exceptions, an overall reduced cerebral network architecture resulting in inadequate information processing [38]. In unilateral CP, there are close relationships between sensorimotor function and the integrity of the white matter of the somatosensory pathways and the corticospinal tract. There are currently hardly any studies on the commissural and association pathways [39]. The thinning of the microstructure and a reduced volume of the thalamocortical projections to the postcentral gyrus in CP with hemiplegia determine the poorer sensorimotor functions due to deficits in sensory information [40]. These network deficits have also been found in spastic CP [41]. The pathomorphologic feature of dyskinetic CP is the reduced integrity of the white matter of the corticospinal tract and the cortico-striatal-thalamocortical connections. These results are also found in the other subtypes of CP [42]. The structural damage pattern depends not only on the severity of the disorder but also on the time of the event.

## 6. CP and Link to Sensorimotor Function and Emotion

This also means that abnormalities can be detected not only in the white and grey matter of the sensory and motor structures, but also in the cerebral structures that are not directly or solely associated with the sensorimotor system [34]. Multisensory processing and cognitive processes are jointly based on the networking of the cortical and subcortical neuronal structures via the microarchitecture of the white matter pathways, through which a constant mutual exchange of information occurs [43]. The clinical result is the cognitive decisions on behavior with the associated emotional regulation. Individuals with dyskinetic CP show an extensive reduction in the volume of white matter and a clearly localized reduction in the connectivity of the parieto-occipital regions and the hippocampus (memory!). The connectivity of the sensory, intraparietal and frontoparietal connections is reduced. Networks that link the integrity of working memory with clinical features are not limited to the structures of the sensorimotor system [41].

Furthermore, in dyskinetic CP, the widespread losses of white matter integrity, which are mainly localized in the parietal lobe, correlate with IQ. Executive performance is related to the microstructure of white matter in the regions that maintain fronto-cortical and posterior cortico-subcortical connections [44]. In addition to gross motor dysfunction, these children exhibit a number of co-morbidities, including epilepsy, visual and gastrointestinal disorders, cognitive impairment, communication deficits and behavioral problems (anxiety, social competence and self-related behavior). The severity of the sensorimotor disability influences the individual’s psycho-physical development, cognitive abilities, quality of sleep and overall quality of life [45]. These results can be taken as evidence of the guiding function of the sensorimotor functions and their development as markers of cognitive-mental and emotional development. There are only 11 studies on the quality of life and health literacy of persons with CP aged between 13 and 38 years in the period 2001 to 2023, with a total of 363 patients [46].

## 7. CP Link to Pain Syndromes

Chronic pain is defined as pain that is present for at least three or up three months and is accompanied by cognitive-mental and physical limitations, and a primary chronic pain disorder is in the ICD-11 labeled as MG30.01. It can be categorized into three phenotypes:

- Nociceptive: Primarily based on peripheral processes in which mechanically, thermally and/or chemically induced afferents are generated (inflammation, degeneration and chronic relative ischaemia) [47];
- Neuropathic: Traumatic or metabolic peripheral and or central nerve damage (grading system according to IASP) [48];
- Nociplastic: Inflammatory maladaptation of the brain characterized by dysfunctional nociception and deficient pain inhibition (grading system according to IASP) [20,21,49,50].

A fourth phenotype results from the combination of the pathologies mentioned.

Chronic nociceptive pain is a characteristic clinical feature of dyskinetic and dyskinetic-spastic CP that is considered a ‘poorly understood co-morbidity’ [13,51]. Spasticity-related pain is more common and more intense than previously thought [52]. The specific phenotype varies greatly in CP adults. Nociceptive (approximately 39%) and nociplastic (approximately 34%) pain account for the largest proportion. Neuropathic pain type is less common [53]. One of the most common localizations of pain is in the feet or lower extremities. In a population of CP patients (5122 people, 58% male, 66% under 18 years of age), 21% had pain in this body region. The risk of developing pain is higher in those with walking ability (GMFS Level I and II), limited ankle mobility and female gender. The risk and intensity of pain also increase with age [54].

The understanding of chronic pain is shifting more and more in the direction of cerebral-nociceptive mechanisms, the development of which is still far from being sufficiently understood. There is evidence of peripheral, spinal and supraspinal mechanisms involving the conscious cognitive area, often combined with symptoms of fatigue syndrome, depression, anxiety and sleep disorders. In many cases, the reaction to visual, gustatory and acoustic stimuli is disproportionate [55,56,57]. The accurate assignment of pain to the phenotypes is very relevant, as the effectiveness of the therapy depends on the underlying pain mechanism, the individual characteristics, the personality, the behavior and the social environment of the patient [58,59].

Due to the intensive directional networking of the brain, sensorimotor function and pain modulation/inhibition are two interlinked functions [13]. Pain modulation is a descending control system for maintaining a functional balance between the inhibitory and inhibitory control circuits of nociceptive processing. Even healthy individuals cannot perform intensive and highly tiring physical exertion without the integration of pain inhibition into the sensorimotor program. During peak athletic performance, the cognitive-evaluative pain component makes it possible so that even with the presence of pain, the performance goal is achieved.

Sensorimotor function and pain cannot be separated as they form a functional unit that has cerebral consequences for the myofascial-skeletal system [60] with impaired muscle function and reduced muscle mass [61,62,63]. In addition, deconditioning due to chronic secondary physical inactivity is the foundation of the pathogenetic chain of chronic degenerative diseases, which can also lead to the development of chronic pain syndromes [34]. People with CP are subject to both a significantly increased risk and a significant 4-fold increase in the incidence of musculoskeletal pain and degenerative diseases. These include myalgia, osteoarthrosis, osteoporosis and, very frequently, sarcopenia, which can be found in children and adults [64]. A study of 15–18-year-old adolescents with CP (*n* = 280) reported 67% acute and 31% chronic pain, with those with acute pain also being 42% chronic pain patients; risk factors for chronic pain are dyskinesia, spastic dyskinesia, bilateral dyskinesia and a severe grade IV and V according to the Gross Motor Function Classification System [14].

At the same time, adolescents very often show deficits in attention, sleep and an overall reduced quality of life. Harvey et al. [65] report in their narrative review that up to 85% of children with CP have pain and that older children and girls with dyskinesia and higher-grade sensorimotor disorders are particularly affected. However, children with mild motor impairments also frequently suffer pain and children with cognitive and communication problems are unable or insufficiently able to communicate their pain. In the case of inadequate communication skills, changes in behavior towards depression and anxiety, neurovegetative symptoms, sleep disturbances, possibly abstinence from food and progressive physical limitations provide indications of a chronic type of pain. The occurrence of psychological abnormalities should draw attention to the development of a nociplastic type of pain. Hypersensitivity and chronic pain are difficult to separate, especially in children with CP who cannot express themselves sufficiently verbally. But both phenomena are closely linked. Spinal sensitization, intensified excitability of nociceptive spinal neurons and altered processing of nociceptive information in the neuromatrix due to neuroplasticity are the mechanisms that cause chronic pain. This central sensitization is preceded by a peripheral sensitization, which can provide disproportionate nociceptive afferents and subsequently cause central sensitization. Spasticity in CP is an involuntary activation of the disinhibited reflex that directly causes localized pain and must initially be separated from chronic pain. Depending on the intensity and extent of the spastic reactions, however, this can also develop into nociplastic pain, which then occurs with extended duration of pain and symptoms of the sensory-discriminative pain component become more intense and widespread and those of the cognitive-emotional component change or are added. Diagnostic instruments must be progressively better adapted to the spectrum of physical, cognitive and communicative limitations and possibilities of CP.

The fact that the intensity of chronic pain restricts physical activities seems logical and easily understood. However, results on the involvement of brain structures of the cognitive-evaluative and affective-emotional pain components are available primarily for the condition of fibromyalgia. The pain status of this clinical picture also includes catastrophizing coping strategies, which are based on a markedly negative attitude towards the pain; this is generally a very common feature of chronic pain [66]. The cognitive distortion ‘catastrophizing’ activates the default mode network (DMN) and, in particular, parts of the ventral and posterior cingulate gyrus (vPCC and dPCC) significantly more intensively in persons with brain damage compared to healthy individuals. The intensity of the pain correlates only with the activity of the dPCC and a subregion to which the sensorimotor functions and cognitive control are attributed [67]. These results can most likely be expected for chronic pain in general. It can also be expected that these brain regions are involved in the pain syndromes of CP and that interactions with other networks are weakened by the structural disorders. One mechanism for the development of functional disorders of pain modulation is persistently or frequently intermittently intensified nociceptive afferents [68]. In the case of existing central damage in CP, the maladaptation causes the pain to persist. The disorders are characteristically linked to catastrophizing, fear of movement and depression, although the evidence is not consistently clear [69,70]. This means that the functional disorder of “chronic pain” can also be traced at the biochemical level through metabolic changes in the cognitive brain structures for self-image and in those for nociceptive information processing. It can be assumed that the chronic pain in CP is additionally supported by the primary structural disorder and is based on comparable changes.

## 8. CP Damage to the Brain Structure and Pathogenesis of Chronic Pain Syndromes

Early structural disorders caused by an inadequate O_2_ supply or other factors directly affect the sensorimotor and subsequently the cognitive-emotional development in pathophysiological terms. The cerebral abnormalities and dysfunctions characterize the adapted deficient peripheral myo-fascial-skeletal structures. This affects the contractile function for mobility. With this development, the periphery also becomes a generator of nociceptive afferents that the brain has to process.

**Figure 6:**
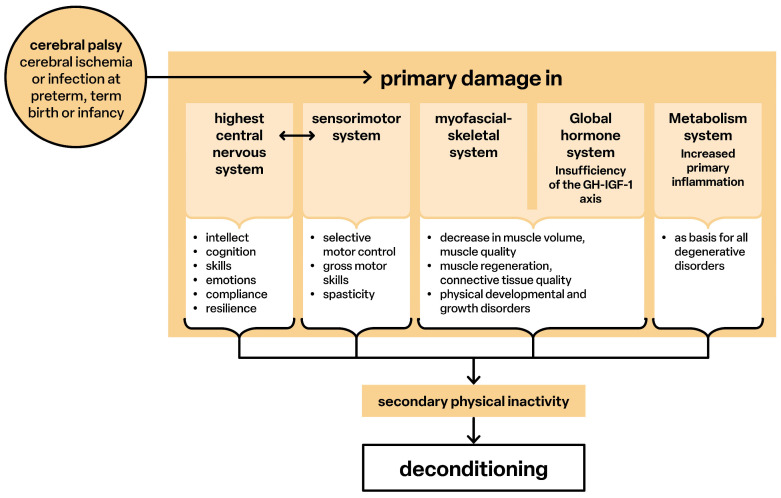
Pathological processes.

Figure 6 provides an overview of the pathological processes in CP. An inadequate blood supply causes primary structural disturbances, the timing and extent of which determine development. Damage to the structures of the somatosensory and corticomotor networks [39] and their interactions leads to loss of selective motor and postural control [71,72] and causes spasticity. Sensorimotor disorders include intellectual, cognitive, emotional functions and communicative behavior [73,74]. Pathological cerebral connectivity also causes deficits in the hypothalamus–pituitary–liver axis (GH–IGF-1; not considered in detail), resulting in growth disorders [75,76]. Muscle mass is qualitatively and quantitatively restricted, and the ability to regenerate is reduced [8,61,62]. Generalized inflammatory processes also occur, and sarcopenia develops at an early stage [13,77,78]. The sensorimotor limitations cause secondary physical inactivity, whereby the already functionally underdeveloped structures develop an ‘extended’ deconditioning. 

The primary disorders are both a predisposing and causal factor of a deficient pain inhibition, and as a result, premature sensitization develops in the periphery and brain, so that persons with CP experience chronic pain.

## 9. Conclusions

Physical, sensorimotor, cognitive-emotional and anti-nociceptive functions depend on the structural and functional integrity of the nervous system. This integrity is the result of the physiological or pathophysiological development of sensorimotor function. An initially healthy nervous system can be impaired at all stages of life by chronic physical inactivity. Inactivity leads to deconditioning as the basis for chronic degenerative diseases, which are initially the cause of chronic secondary pain and from which chronic primary nociplastic pain may develop. In CP, damage to the brain and delayed sensorimotor function directly affect cognition and emotional regulation. This results in children experiencing issues in learning, behavior, language development and school performance since sensorimotor function and high-performing cerebral abilities are interlinked and interdependent. These factors combined result in a secondary physical inactivity, which may turn into deconditioning. Therapy interventions promote development and counteract the deconditioning process. This is crucial to improve movement, cognition and emotional control as well as the reduction of pain syndromes in children and adults with CP.

## Data Availability

No new data were created.

## Abbreviations

The following abbreviations are used in this manuscript:

CP	Cerebral palsy
fMRI	Functional magnetic resonance imaging
CNS	Central nervous system
SMS	Sensorimotor system
